# Non-Uniform Corrosion Monitoring of Steel Pipes Using Distributed Optical Fiber Sensors in the Fluctuation Zone of a Coastal Wharf

**DOI:** 10.3390/s25103194

**Published:** 2025-05-19

**Authors:** Jiguo Chen, Ruiqi Zhang, Qianwu Li, Hongke Wang, Qiangqiang Ma, Qi Fan, Liang Fan, Zequan Lin

**Affiliations:** 1 Suzhou Nuclear Power Research Institute, Suzhou 215004, China; 15850086717@163.com (J.C.); liqianwu@cgnpc.com.cn (Q.L.); wanghongke@cgnpc.com.cn (H.W.); 2 Key Laboratory of Advanced Marine Materials, Institute of Oceanology, Chinese Academy of Sciences, Qingdao 266071, China; zhangruiqi232@mails.ucas.ac.cn (R.Z.); jackmqq@163.com (Q.M.); yc27982@connect.um.edu.mo (Q.F.)

**Keywords:** distributed optical fiber sensors, non-uniform corrosion monitoring, coastal wharf, steel pipe

## Abstract

Steel pipes, while essential for modern infrastructure due to their high strength and load-bearing capacity, are prone to corrosion in the marine environment, leading to material degradation, compromised structural integrity, and elevated safety risks and economic losses. In this study, distributed fiber-optic sensors were deployed on steel pipe surfaces to monitor corrosion in the splash zone (a region particularly vulnerable to cyclic wet–dry conditions). The sensors were engineered to withstand aggressive marine exposure. Strain variations induced by expansive corrosion products were detected via the fiber-optic array and used to calculate localized mass loss. Color-coded corrosion severity maps were generated to visualize the non-uniform corrosion distribution. Experimental results demonstrate that sensor-derived mass loss values align with 3D laser scanning measurements, validating the operational efficacy of distributed fiber-optic sensing for marine corrosion monitoring. This approach provides quantitative insights into the field applicability of optical sensing in structural health monitoring.

## 1. Introduction

Steel pipes are fundamental components of modern infrastructure, widely employed in marine environments such as coastal bridges, offshore platforms, and other critical structures owing to their high strength, durability, and load-bearing capacity [1]. However, these structures are inherently vulnerable to corrosion, particularly when exposed to aggressive environments such as seawater and humid atmospheres [2]. Corrosion is an electrochemical process that leads to the gradual degradation of steel, resulting in material loss, a reduced cross-sectional area, and, ultimately, a decline in structural performance [3]. Left unchecked, corrosion can compromise the safety, serviceability, and lifespan of steel-pipe-supported infrastructure, posing significant economic and safety risks [1,4].

Traditional methods for monitoring corrosion in steel pipes include visual inspections, electrochemical techniques (e.g., potentiodynamic polarization and electrochemical impedance spectroscopy), and non-destructive testing methods such as ultrasonic testing and radiography [5]. While these techniques have proven useful, they are often limited by their localized nature, providing data only at discrete points or designated fixed monitoring points [6]. Furthermore, these methods are typically labor-intensive, time-consuming, and costly, particularly for large-scale or hard-to-reach structures such as offshore platforms or submerged steel pipes [7]. As a result, there is a pressing need for innovative monitoring solutions that can provide continuous, real-time, and spatially distributed data on corrosion progression [8].

Distributed optical fiber sensors (DOFSs) have emerged as a transformative tool for structural health monitoring, leveraging light scattering principles (e.g., Rayleigh, Brillouin, or Raman scattering) and wavelength shifts to measure strain, temperature, and vibration along the entire length of an optical fiber with a high spatial resolution (down to millimeters) and long-distance coverage (tens of kilometers) [9]. This technology offers unparalleled advantages for large-scale infrastructure, such as steel pipes, bridges, pipelines, and offshore platforms, where traditional point sensors—limited to discrete measurements—fail to capture continuous, real-time, and spatially distributed data [10]. By providing a full spatial profile of structural parameters, DOFSs enable the detection of localized anomalies (critical for early-stage corrosion monitoring, which often initiates at specific points) and the assessment of global structural behavior, while their immunity to electromagnetic interference and chemical inertness enhance their suitability for challenging applications [11]. The high spatial resolution, adjustable from millimeters to meters depending on the technique, allows for precise identification of corrosion hotspots and subtle material changes over time [12]. Furthermore, DOFSs eliminate the need for extensive wiring and the complex data acquisition systems required by conventional sensors, making them a scalable and efficient solution for long-term monitoring of corroded structures [13].

Recent studies have demonstrated the potential of DOFSs for detecting corrosion-induced strain changes in steel structures. For instance, researchers have successfully used distributed strain sensing to monitor the degradation of reinforced concrete structures, where the corrosion of embedded steel rebar leads to measurable strain variations [14,15]. Similarly, DOFSs have been applied to monitor the structural integrity of pipelines and offshore structures, where corrosion is a major concern [16]. Sun et al. employed long-gauge fiber Bragg grating (LG-FBG) and long-gauge Brillouin optical time-domain analysis (LG-BOTDA) sensing systems to monitor structural deformations in pipelines. The feasibility and accuracy of this approach under complex loading conditions were validated through full-scale pipeline loading experiments [17]. Tan et al. utilized distributed optical fiber sensors (DOFSs) to monitor the response behavior of pipelines subjected to interactive bending and denting deformations in real time. A numerical model was developed to assess deformation effects under varying load conditions [18]. Furthermore, Tan et al. installed distributed optical fibers on low-carbon steel and stainless steel pipes, which were subjected to accelerated corrosion in a 3.5% sodium chloride solution. The strain distribution measured by optical frequency domain reflectometry (OFDR) was used to model corrosion-induced mass loss and enable early warnings of corrosion damage [19]. Shen et al. simulated a corrosive marine environment and conducted accelerated corrosion experiments on steel pipes with surface-mounted distributed optical fiber sensors [6]. By continuously monitoring the evolution of surface strain, a model for non-uniform corrosion-induced mass loss was established, and the corresponding volumetric expansion coefficient associated with corrosion was determined. These advancements highlight the feasibility of adapting DOFSs for monitoring steel pipes, where early detection of corrosion could significantly enhance maintenance strategies and extend the service life of infrastructure.

The above studies demonstrate the feasibility of optical fiber sensing technologies for corrosion monitoring under controlled laboratory conditions, particularly in terms of strain detection and mass loss modeling. However, significant challenges remain in translating these findings to real-world marine infrastructure. One key issue is the long-term stability and reliability of optical fiber sensors under harsh oceanic conditions characterized by high humidity, salinity, and biofouling. Effective installation and encapsulation techniques are critical to ensure sensor durability and data acquisition accuracy. Moreover, the applicability and accuracy of corrosion monitoring models developed in laboratory settings are still uncertain when exposed to complex real-world phenomena such as non-uniform corrosion. These factors may influence the interpretation of sensing data and limit the generalizability of the models. Therefore, further research is needed to adapt sensor systems for marine environment deployment and establish long-term validation through in situ data collection.

In this study, distributed optical fiber sensors were deployed on the surface of steel pipes to monitor corrosion occurring in the splash zone (a region particularly vulnerable to corrosion due to fluctuating wetting and drying conditions). The sensor was carefully engineered to withstand the aggressive marine environment. To comprehensively evaluate the strain response of the sensors under different corrosion protection scenarios, three protective conditions were considered: an intact corrosion protection wrapping system, a partially damaged corrosion protection wrapping system, and bare steel without a corrosion protection wrapping system. The corrosion-induced strain from the optical fiber sensors was recorded periodically for 162 days. Based on the deducted mass loss calculation model, the mass loss of the steel pipes was obtained through the strain measured from the sensors, which was validated from the 3D scanner afterwards. Finally, a heatmap illustrating the variation in mass loss over time was obtained to visualize the non-uniform corrosion and the localized severity of the corrosion.

## 2. Experimental Design

**Figure 1:**
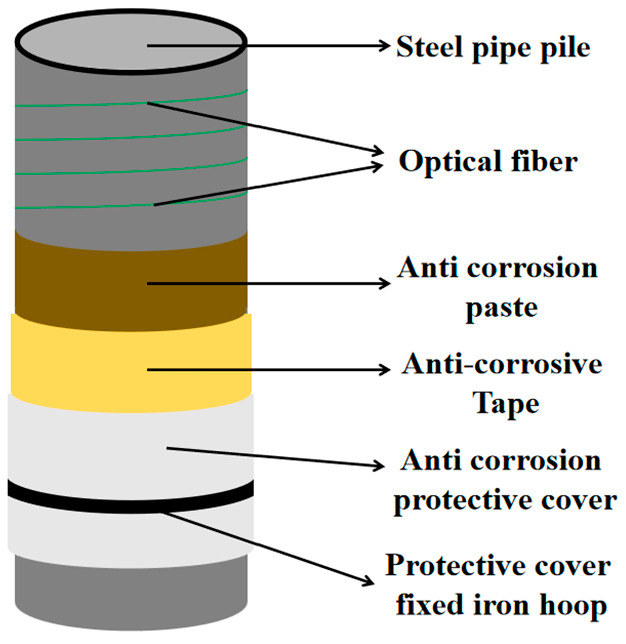
Structural diagram of the steel pipe with distributed optical fiber sensors and the mineral encapsulation coating system.

The main purpose of the experiment was to locate the corroded area and determine the degree of corrosion when non-uniform corrosion occurred on the steel pipe surface. Considering that, in actual structures, steel pipes are not exposed to seawater but undergo anti-corrosion treatment, we used mineral encapsulation coating technology to protect steel pipes from corrosion. Optical fibers were spirally wrapped around the pipe surface and additionally adhered at selected points for secure fixation before the mineral encapsulation coating system was installed, as shown in Figure 1. The mineral encapsulation coating system included an anti-corrosion paste layer, an anti-corrosion tape layer, an anti-corrosion protective cover layer, and an outermost fixed iron hoop in sequence from inside to outside.

### 2.1. Materials

The steel pipe had an outer diameter of 110 mm, a wall thickness of 5 mm, and a height of 250 mm. The mineral encapsulation coating system was purchased from Qingdao Diante New Material Technology Co., Ltd., Qingdao, China. A single-mode optical fiber with a diameter of 0.88 mm was used as the distributed optical fiber sensor. The optical fiber was composed of a fiber core, cladding, an inner coating, and an outer coating. The fiber core and cladding were made of silica, and the inner and outer coatings were made of acrylic resin, which provided mechanical protection for the fiber core and cladding.

### 2.2. Mineral Encapsulation Coating System Installation

**Figure 2:**
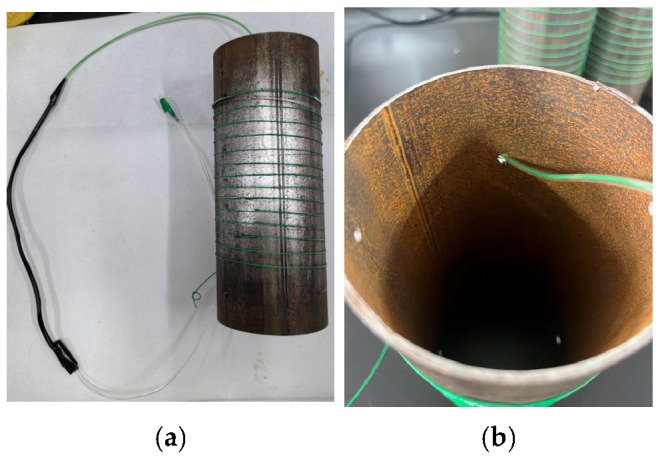
(**a**) Optical fiber deployment and mechanical protection; (**b**) insertion of the fiber end inside the steel pipe for further mechanical protection.

The mineral encapsulation coating system was implemented through a multi-step process beginning with abrasive blasting of the steel pipe to remove surface rust. A single-mode optical fiber was helically wound around the pipe with 15 turns at a 10 mm pitch, followed by fusion-splicing the fiber end to an LC/APC connector (<0.05 dB loss). The splice joint was encapsulated using a dual-layer protective system. A fluoropolymer heat-shrink tube (1.5 mm inner diameter) was thermally fused at 110 °C (±0.5 °C) for 120 s and then armored with a UV-resistant PET sleeve (2.5 mm ID), achieving IP68-rated sealing as shown in Figure 2a. A 2 mm access port was drilled into the pipe, and the reinforced fiber assembly was inserted inside for further mechanical protection. This process ensured corrosion-resistant mechanical protection in coastal environments, providing reliable strain monitoring for non-uniform corrosion detection.

**Figure 3:**
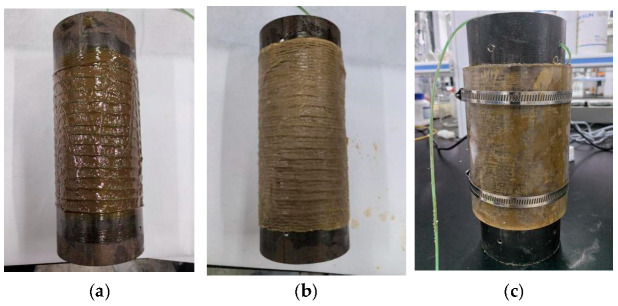
(**a**) Steel pipes coated with anti-corrosion paste; (**b**) steel pipe with anti-corrosion tape; (**c**) fixing of the acrylic protective cover on the steel pipe with iron hoops.

Next, anti-corrosion paste was applied to the steel pipe surface evenly with a brush, and the thickness of the anti-corrosion paste was 200–250 μm. The steel pipe surface after the application of the anti-corrosion paste is shown in Figure 3a. Then, anti-corrosion tape was tightly wrapped around to prevent the loss of anti-corrosion paste. Lastly, an acrylic protective cover was tightly wrapped around the steel pipe surface and fixed with iron hoops to provide further mechanical protection.

**Figure 4:**
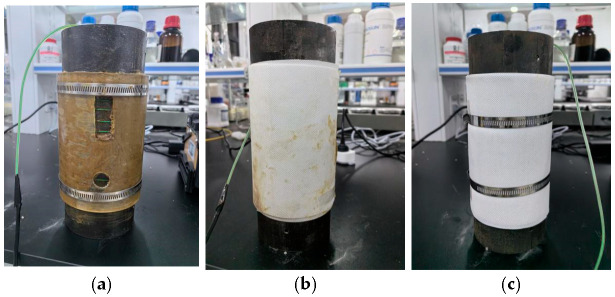
(**a**) Artificial holes to simulate damage to the mineral encapsulation coating system; (**b**) the steel pipe coated with anti-corrosion paste and covered with non-woven fabric; (**c**) bare steel covered with non-woven fabric without anti-corrosion protection.

For comparison, three other control groups were designed. For the first group, in the longitudinal direction of the steel pipe, with two skylights, one large hole and one small hole on the acrylic protective cover were made to simulate protection damage. This was mainly to study whether the corrosion would expand around the damaged area with the erosion of sea waves after localized damage, as shown in Figure 4a. For the second group, anti-corrosion paste was first applied to the steel pipe surface, which was then wrapped with non-woven fabric. This was mainly used to investigate the corrosion protection performance when the outer protective cover falls off, as shown in Figure 4b. For the third group, non-woven fabric was directly wrapped around the steel pipe to protect the optical fiber sensors without using the mineral encapsulation coating system, as shown in Figure 4c.

### 2.3. Site Installation

**Figure 5:**
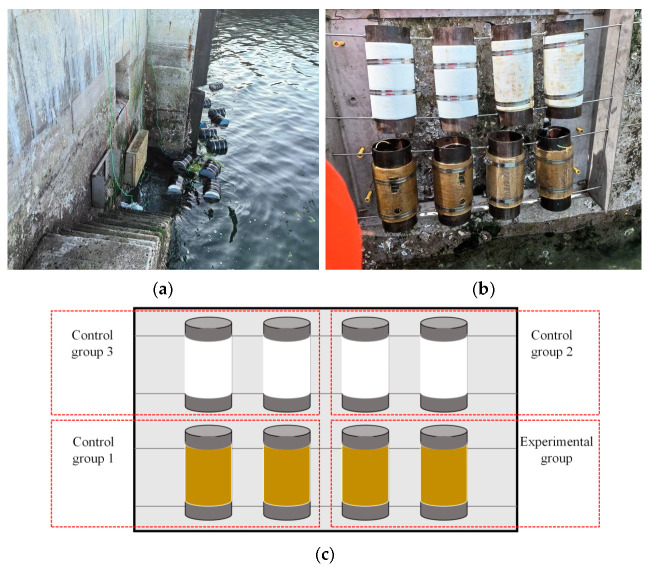
Test Site. (**a**) Surrounding environment; (**b**) on-site specimen fixation; (**c**) schematic diagram of steel pipe installation.

The experimental site was the Xuejiadao Port Test Site in Qingdao, and the surrounding environment of the site is shown in Figure 5a. The stainless steel frame was fixed on the concrete wall with expansion screws. The next step was to thread the steel pipe with steel wire and hang it on the steel frame following the sequence of the experimental group, the first control group, the second control group, and the third control group as shown in Figure 5c. Subsequently, stainless steel mesh was applied to cover the steel frame for collision avoidance.

### 2.4. Monitoring Principle

**Figure 6:**
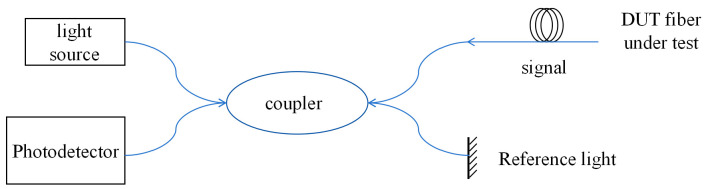
Basic principle of OFDR.

The monitoring instrument used was the “OSI-D” distributed optical fiber data acquisition device from Wuhan Haoheng Technology, with a sensing length of 100 m, a sampling rate of 100 Hz, a spatial resolution of 0.64 mm, strain repeatability of ±4 με, and a strain measurement range of ±12,000 με. The basic monitoring principle of this device is based on a combination of optical frequency domain reflection (OFDR) technology and optical heterodyne detection technology as shown in Figure 6. The continuous light emitted by the light source in linear scanning is divided into two paths by the coupler, one of which serves as the reference light and the other as the detection light emitted into the fiber under analysis. When the probe light propagates forward in the optical fiber, it continuously generates Rayleigh scattering signals. The signal light and the reflected reference light pass through the coupler, undergo beat frequency interference, and are detected by the photodetector.

To perform strain or temperature sensing, the Rayleigh scattering signals from a reference state (baseline measurement) and a measurement state (perturbed by strain/temperature) were analyzed. The signals were segmented into multiple discrete intervals (signal windows) based on the spatial resolution of the sensing system. For each window, the spectral shift of the scattered light was calculated using a spectral analysis method. The magnitude of this spectral shift was then correlated with the corresponding strain or temperature change at that location by applying a pre-determined frequency shift coefficient, which accounts for the fiber’s material properties and environmental sensitivity. This process enables distributed sensing, providing continuous, spatially resolved measurements of strain or temperature along the fiber. By precisely measuring these frequency shifts, the localized strain or temperature distribution along the entire fiber can be calculated based on Equation (1):

(1) $$\frac{\Delta \lambda }{\lambda }=\frac{\Delta \nu }{\nu }=K_{T}T+K_{\varepsilon }\varepsilon$$

where λ and υ are the mean optical wavelength and frequency, respectively, and *K_T_* and *K_ε_* are the temperature and strain sensitivity coefficients, respectively. At a constant temperature, the spectral shift is dependent on the strain.

## 3. Results and Discussion

### 3.1. Micro-Strain Response Induced by Corrosion of Steel Pipes

**Figure 7:**
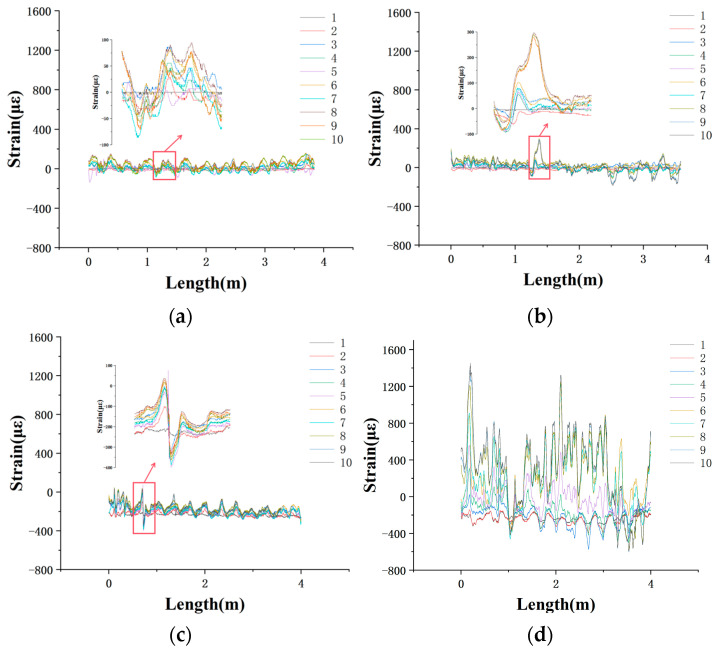
(**a**) Strain variation along the length of the distributed optical fibers over ten measurement cycles for the steel pipes in the (**a**) experimental group, (**b**) first control group, (**c**) second control group, and (**d**) third control group.

Corrosion monitoring of steel pipes was conducted during the low-tide period over ten measurement cycles, with intervals ranging from 16 to 17 days throughout the study. Due to the high continuity requirements of distributed optical fiber sensing systems, any breakage in the sensing fiber necessitates the re-establishment of a new reference signal. Although protective measures were implemented at the fiber outlet interfaces, multiple breakages still occurred under the influence of waves and other environmental factors. As a result, some datasets were incomplete or exhibited temporal inconsistencies. To ensure the accuracy and reliability of the analysis, one representative specimen with complete and well-responding monitoring data was selected from each protective configuration for graphical presentation. The strain–time response curves for the four specimen types are shown in Figure 7. For each processed dataset, the strain induced by temperature variations was compensated for in the corrosion-induced strain analysis, and the local temperature was recorded at the testing site during each measurement.

For the specimen in the experimental group, the strain increased steadily throughout the experimental period. In the initial stage, the strain remained at a relatively low level and gradually accumulated over time, eventually stabilizing at approximately 100 με without exhibiting any abrupt changes. This behavior indicates that the steel pipe was barely corroding, which can be attributed to the synergistic protection effect of the anti-corrosion paste layer, the anti-corrosion tape layer, and the anti-corrosion protective cover layer. The anti-corrosion paste formed a dense protective film on the surface of the steel pipe, effectively isolating corrosive media and suppressing electrochemical reactions. Meanwhile, the protective cover layer provided mechanical protection and served as an additional barrier, preventing the intrusion of particulate impurities and biofouling, thereby reducing physical damage and slowing down the rate of corrosion. One thing that needs to be mentioned is that the 15 distinct peaks exhibited in the strain curve are likely associated with localized stress concentrations caused by the periodic impact of ocean waves. The number of peaks closely matched the number of fiber coil turns, suggesting that transient dynamic loads significantly influenced the stress on the fiber’s sensitive regions.

In the early stages of the experiment, the specimen in the first control group exhibited a gradual increase in strain, similar to the trend observed in the experimental group. However, in the middle to later stages, a localized marked rise in strain was observed in the perforated regions, indicating accelerated corrosion due to a localized protective failure. Although this specimen was also equipped with a triple-layer protection system, the perforations served as direct pathways for corrosive media, substantially compromising the protective efficacy. Under frequent tidal fluctuations and wave impacts, seawater continuously penetrated the perforations, intensifying localized corrosion. The orientation of the openings toward incoming waves further elevated the local dynamic pressure, exacerbating the corrosion severity. Additionally, anti-corrosion paste may have been lost through the perforations, leading to further degradation of the protective capability. The rapid increase in strain suggests that localized corrosion had propagated into adjacent regions, demonstrating that even minor structural defects such as perforations can critically impair the overall service performance. This result underscores the crucial role of localized protective integrity in ensuring the long-term durability of structures in marine environments.

The strain observed in the specimen in the second control group remained predominantly negative throughout the experiment. Although fluctuations occurred over time, no clear trend of cumulative increase was detected, indicating the absence of significant corrosion-related structural degradation. The negative strain signals are likely attributed to non-corrosive factors such as coating detachment or redistribution. Compared with specimens in the experimental group and first control group, which employed a triple-layer protection system, the specimen in the second control group lacked the anti-corrosion tape layer and the anti-corrosion protective cover layer, relying solely on the anti-corrosion paste, thereby offering a moderate level of protection. After the experiments, the non-woven fabric was peeled off, and residual anti-corrosion paste and an absence of visible corrosion on the steel surface were observed, suggesting that the coating still provided an effective barrier. However, the absence of mechanical protection made the coating more vulnerable to environmental influences such as wave-induced impacts that could take away the anti-corrosion pate and result in negative strain in the long run. Although the current extent of corrosion was minimal, prolonged service exposure may lead to localized protective failure if anti-corrosion paste loss continues. It is therefore recommended that future engineering applications enhance the coating’s adhesion and consider incorporating a secondary protective layer to improve long-term durability.

The strain in the specimen in the third control group increased rapidly from the onset of the experiment, with a cumulative rate significantly higher than that of the other specimens, indicating the poorest protective performance among all tested configurations. Due to the absence of an anti-corrosion paste and tape, the steel surface was directly exposed to seawater, where electrochemical corrosion processes were promptly initiated. The combined effects of oxygen concentration cell formation and chloride ion penetration further accelerated the corrosion rate. Although the non-woven fabric provided a limited physical barrier, it was insufficient to prevent the ingress of corrosive agents and could not withstand wave scouring or the cyclical wet–dry conditions that facilitate the detachment of corrosion products. Repeated dynamic pressure from wave action not only promoted the flaking of corrosion products and the continuous exposure of fresh metal surfaces but also intensified stress disturbances within the pipe surface, leading to persistent and rapid strain accumulation. Corrosion-induced damage became inevitable, severely compromising the long-term service performance of the structure.

**Figure 8:**
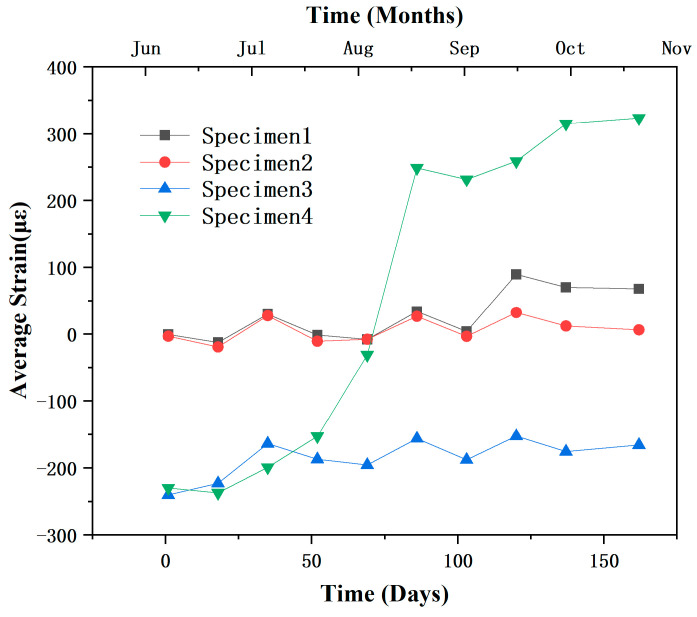
The average strain of each specimen as a function of time.

The experimental results from Figure 8 demonstrate that the integrity of the protective system is a critical factor in determining the strain accumulation rate and durability of steel pipes in marine environments. Specimen 1, equipped with a complete protection system comprising an anti-corrosion paste layer, an anti-corrosion tape layer, and an anti-corrosion protective cover layer, exhibited the slowest strain growth. Although Specimen 2 also featured a three-layer protection system, the presence of localized perforations compromised its sealing capability, resulting in accelerated localized corrosion and a rapid increase in strain. Specimen 3, which lacked the anti-corrosion tape layer, showed no significant corrosion, but anti-corrosion paste loss led to fluctuating strain responses, indicating uncertainty in long-term performance. Specimen 4, which only had the non-woven fabric for optical fiber sensor protection, experienced the most severe corrosion and the highest strain accumulation rate.

Furthermore, the experiment spanned the summer and autumn seasons, during which environmental temperature variations significantly influenced corrosion behavior. Elevated temperatures in June and August intensified corrosion reactions, resulting in corrosion-induced strain growth, particularly in specimens with weaker protection. High temperatures may also reduce coating adhesion, increasing the risk of a localized failure. From September to November, lower temperatures slowed corrosion progression and reduced strain accumulation rates. Overall, the period from June to November revealed the pronounced effectiveness of the mineral encapsulation coating system in preventing corrosion, highlighting its role in effectively blocking corrosive media and reducing steel degradation. In contrast, unprotected specimens experienced more severe corrosion, especially in areas subject to strong wave impacts. In engineering practice, particular attention should be paid to the integrity and weather resistance of protective systems. The monitoring and timely repair of localized defects are essential to ensure the long-term serviceability and prevent corrosion of marine infrastructure.

### 3.2. Verification of Experimental Results

In this experiment, a quantitative analysis of the corrosion process was achieved by monitoring strain variations on the steel pipe surface using distributed optical fiber sensors in conjunction with a non-uniform corrosion-induced mass loss model. The method for estimating mass loss via strain measurements is introduced as follows [6].

Assume that uniform corrosion occurs at a measurement point in a distributed optical fiber system. The original radius of the steel pipe before corrosion is R_0_. The steel pipe reacts with environmental elements such as oxygen, water, and ions to form corresponding oxides. The insoluble corrosion products adhere to the surface of the steel pipe. The radius measured by the optical fiber, which includes the rust layer, is R_c_, while the radius after removing the rust layer is R_w_.

The height of the unit is related to the arrangement of the optical fiber. If the fiber is arranged in a non-uniform helical pattern, the height is half the sum of the distances from the fiber to the ones directly above and below it. If the fiber is arranged in a uniformly spaced helical pattern, the height equals the spacing between two adjacent fibers.

The angular size can be determined by the radius of the steel pipe and the arc length of its outer surface, which is related to the system’s sampling resolution. The expression is as follows:

(2) $$\alpha =\frac{L_{1}}{2\pi R_{0}}.2\pi =\frac{L_{1}}{R_{0}}$$

Therefore, the volume of the corroded steel within any unit on the surface of the steel pipe (V_P1_) and the volume of the rust products (V_r1_) formed can be respectively expressed as:

(3) $$V_{p1}=\frac{\alpha }{2\pi }.\pi h[{\left(R_{0}\right)}^{2}-{\left(R_{W}\right)}^{2}]=\frac{\alpha h}{2}[{\left(R_{0}\right)}^{2}-({R_{W}}^{2})]$$

(4) $$V_{r1}=\frac{\alpha }{2\pi }.\pi h[{\left(R_{c}\right)}^{2}-{\left(R_{W}\right)}^{2}]=\frac{\alpha h}{2}[{\left(R_{c}\right)}^{2}-{\left(R_{W}\right)}^{2}]$$

Then, the mass loss of the steel pipe within the unit due to corrosion can be expressed as:

(5) $$\Delta m_{i}^{j}=V_{p1}\rho =\frac{\alpha h\rho }{2}[{\left(R_{0}\right)}^{2}-{\left(R_{W}\right)}^{2}]$$

The increase in pipe diameter leads to an increase in the tensile strain measured by the circumferential strain fiber. If the change in strain is $\varepsilon_{i}^{j}$, then the radius of the steel pipe after corrosion, including the rust layer R_c_, is:

(6) $$\varepsilon_{i}^{j}=\frac{L_{2}-L_{1}}{L_{1}},R_{c}=R_{0}\left(1+\varepsilon_{i}^{j}\right)\;\;\varepsilon_{i}^{j}>0$$

(7) $$\varepsilon_{i}^{j}=\left|\frac{L_{2}-L_{1}}{L_{1}}\right|,R_{c}=R_{0}\left(1-\varepsilon_{i}^{j}\right)\;\;\varepsilon_{i}^{j}<0$$

The mass loss due to corrosion, denoted as ∆m, can be calculated using the following equation:

(8) $$\Delta m_{1}=\sum_{j=1}^{m}\sum_{i=1}^{n}\frac{L_{1}h\rho R_{0}}{2\left(\lambda -1\right)}\left[{\left(\varepsilon_{i}^{j}\right)}^{2}+2\varepsilon_{i}^{j}\right],\varepsilon_{i}^{j}>0$$

(9) $$\Delta m_{2}=\sum_{j=1}^{m}\sum_{i=1}^{n}\frac{L_{1}h\rho R_{0}}{2\left(\beta -1\right)}\left[{\left(\varepsilon_{i}^{j}\right)}^{2}-2\varepsilon_{i}^{j}\right],\varepsilon_{i}^{j}<0$$

During the calculation process, micro-strain data and their distribution along the fiber deployment path on the steel pipe surface were first extracted. These values, together with the geometric parameters of the pipe and the sensing length, were substituted into the mass loss model. The model parameters λ and β were adopted from [6] and set to λ = 1.35 and β = 0.97, respectively. By incorporating the fiber-measured micro-strain data into the model, the calculated mass loss of the steel due to corrosion during the target period was determined to be 52.1566 g.

**Figure 9:**
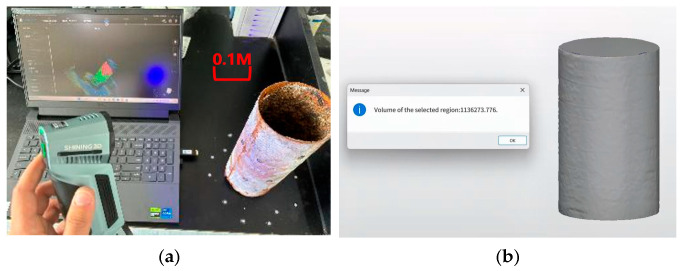
(**a**) The scanning process using the 3D scanner; (**b**) the corroded volume of the steel pipe calculated from the 3D scanner.

To validate the reliability of the corrosion mass estimation method based on strain data, we employed three-dimensional laser scanning technology to conduct high-precision modeling and comparative analysis of the geometric morphology of the steel pipes before and after corrosion as shown in Figure 9a. The three-dimensional scanner used in the experiment had a scanning accuracy of 0.02 mm, a volumetric accuracy of 0.015 + 0.035 mm/m, and a spatial point distance of 0.05 mm. The scanning speed was no less than 5,370,000 points per second, with a maximum static scanning area of at least 650 mm × 550 mm and a scanning depth of field of 550 mm. Considering that internal wall corrosion and biofouling may influence the measurement results, the external cylindrical volume method was consistently used in this study to assess the material loss caused by corrosion.

Based on the scanning data, the external cylindrical volume of the steel pipe after corrosion was determined to be approximately 1136.27 cm^3^ in Figure 9b. By estimating the volume loss through comparison with the theoretical volume before corrosion and multiplying by the steel density, the corrosion mass loss was calculated to be 56.06 g, which is about 7.48% higher than the result obtained using the optical fiber strain-based method. This discrepancy may arise from the different sensitivities of the two methods to corrosion. The three-dimensional scanning technique is more sensitive to localized geometric changes such as small pits and surface roughness, making it more responsive to early-stage, minor corrosion. In contrast, distributed optical fiber strain monitoring primarily exhibits a weaker response to minor surface corrosion-induced morphological changes. Despite the relative error, the numerical trends and magnitudes of the corrosion mass loss estimated by both methods are highly consistent, providing strong evidence that the strain inversion method possesses good accuracy and practical applicability in engineering contexts.

Through cross-validation with the three-dimensional scanning results, the credibility of the corrosion mass estimation method based on strain data was significantly enhanced. This also provides a practical basis for integrating optical fiber sensing and geometric measurement technologies, thus paving the way for the development of a multidimensional corrosion monitoring system. In summary, the experiment validated the effectiveness of the corrosion mass loss assessment method based on distributed optical fiber micro-strain inversion. This provides a theoretical foundation and technical support for accurate corrosion state identification and early warning systems.

### 3.3. Spatial Characteristics of the Corrosion Distribution

**Figure 10:**
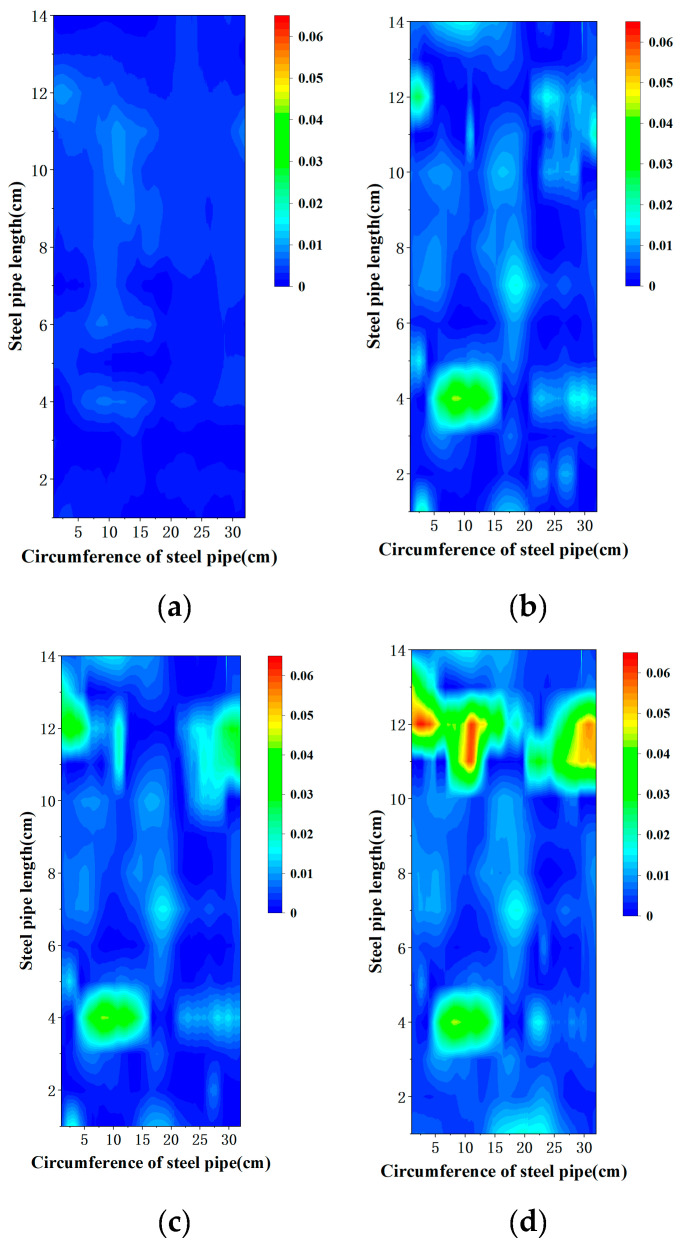
Non-uniform corrosion-induced mass loss (g) distribution on the surface of the fourth specimen on the (**a**) 35th day; (**b**) 86th day; (**c**) 120th day; (**d**) 162nd day.

We employed distributed optical fiber sensing technology to achieve high-resolution, full-coverage, and dynamic monitoring of the corrosion process of steel pipes in a tidal marine zone environment. Quantitative analysis focused on Specimen 4, which exhibited most significant corrosion. Four sequential mass loss heatmaps (Figure 10) captured the spatiotemporal evolution of corrosion, demonstrating progressive localized degradation. The heatmap was generated by processing strain data collected from distributed optical fiber sensors helically wrapped around the steel pipe’s outer surface. Each helical loop of the fiber contains approximately 500 sensing points, with the spatial distribution allowing each loop’s data to correspond to a row in the heatmap. Micro-strain values from these sensing points were input into the corrosion mass loss model (Equations (8) and (9)) to calculate the localized corrosion-induced mass loss at each position, thereby constructing a two-dimensional distribution of non-uniform corrosion across the pipe surface. The computed mass loss data in matrix format were visualized using a contour map in Origin software, where the color scale explicitly represents corrosion mass loss values in grams (g).

In the initial stage (Figure 10a), the heatmap predominantly appears deep blue, indicating that corrosion has not yet occurred extensively. Only slight strain fluctuations can be observed in isolated regions, which may correspond to the emergence of early micro-corrosion points. As time progresses (Figure 10b), these initial corrosion points gradually expand and propagate along the axial direction of the fiber, forming continuous light-blue band-like areas. Some regions show orange high-strain zones, suggesting the intensification of local corrosion and the initial stages of corrosion spreading. In the middle to later stages (Figure 10c), the corrosion area further extends toward the upper half of the steel pipe, with increased light-green and orange regions, indicating that environmental factors are impacting a broader area and the corrosion severity is deepening. During this phase, the periodic immersion and exposure caused by tidal effects likely enhanced the accumulation of corrosive agents in seawater, accelerating corrosion development. In the final stage (Figure 10d), the heatmap shows several originally discrete high-strain corrosion zones merging, forming a continuous distribution of green to orange-red corrosion bands. Some areas appear red, with significant mass loss peaks, reflecting severe localized corrosion. These high-corrosion zones are concentrated in regions with substantial material loss, indicating that these areas, long exposed to the cyclic “dry–wet–oxygen–salt” conditions, are high-sensitivity zones for corrosion development.

**Figure 11:**
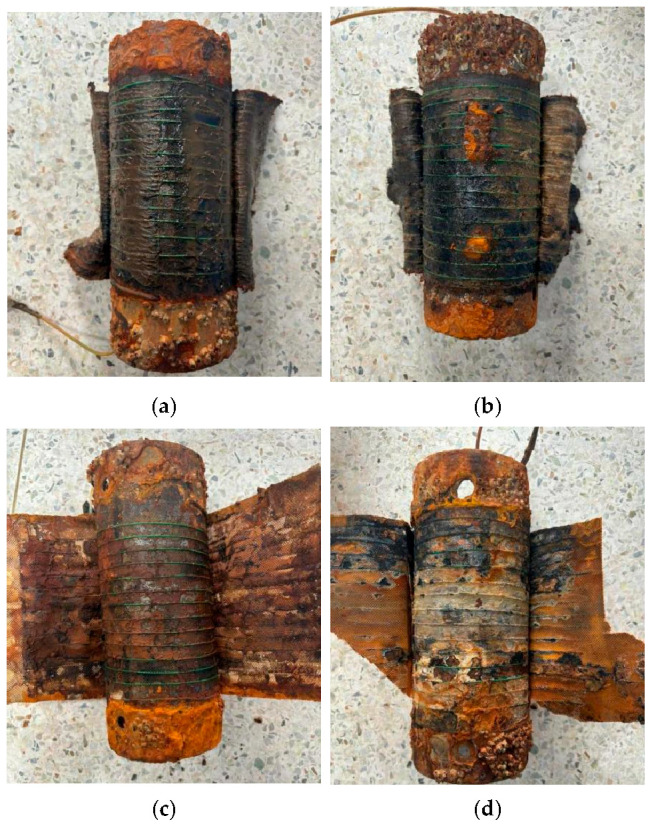
Appearance of the steel pipe surface of the (**a**) first specimen, (**b**) second specimen, (**c**) third specimen, and (**d**) fourth specimen after removing the anti-corrosion protection system.

Figure 11 shows the appearance of the steel pipe after removing the anti-corrosion protective cover layer and peeling off the anti-corrosion tape layer. Anti-corrosion paste was visible on the steel pipe surfaces in the first and second specimens except at the damaged location in the second specimen. The thickness of the anti-corrosion paste was nearly identical in the first and second specimens but reduced significantly in the third specimen. Severe corrosion was observed across the surface of the fourth specimen. These observations align with the strain measurement results obtained from the optical fiber sensors.

## 4. Conclusions

This study utilized a distributed fiber-optic sensing system to monitor the non-uniform corrosion of steel pipes in coastal wharf fluctuation zones. The key findings can be summarized as follows:

- The mineral encapsulation coating system effectively prevented corrosion throughout the 162-day experiment, even when only anti-corrosion paste was applied to the surface. Uncoated specimens exhibited non-uniform surface corrosion;
- The fiber-optic sensors demonstrated robust performance during the test period, successfully capturing corrosion initiation and propagation dynamics (spatial resolution, 5 mm), validating the installation methodology;
- Optical fiber sensor-derived mass loss calculations yielded 52.16 g (±0.8 g), while 3D scanning measurements showed 56.06 g (±0.5 g), indicating a 7.48% inter-method discrepancy. This establishes optical fiber sensing as a viable complement to conventional non-destructive testing techniques;
- The mass loss distribution on the steel pipe surface was visualized in color cloud maps, and non-uniform corrosion and severe localized corrosion were clearly observed, illustrating that the distributed optical fiber sensors can be applied to monitor steel pipe corrosion in real coastal environments;
- Future work will focus on enhancing sensor durability in aggressive environments and integrate machine learning models to predict the remaining service life.

## Data Availability

All relevant data will be made available upon request.
